# Facilitators and Barriers for Successful Implementation of Interconception Care in Preventive Child Health Care Services in the Netherlands

**DOI:** 10.1007/s10995-016-2046-5

**Published:** 2016-07-06

**Authors:** Meertien K. Sijpkens, Eric A. P. Steegers, Ageeth N. Rosman

**Affiliations:** Department of Obstetrics and Gynecology, Division of Obstetrics and Prenatal Medicine, Erasmus MC, PO Box 2040, 3000 CA Rotterdam, The Netherlands

**Keywords:** Interconception care, Maternal and child health services, Pediatric care, Implementation

## Abstract

*Objectives* Successful implementation of preconception and interconception care contributes to optimizing pregnancy outcomes. While interconception care to new mothers could potentially be provided by Preventive Child Health Care services, this care is currently not routinely available in the Netherlands. The purpose of this study was to identify facilitators and barriers for implementation of interconception care in Preventive Child Health Care services. *Methods* We organized four focus groups in which Preventive Child Health Care physicians and nurses, related health care professionals and policymakers participated. A semi-structured interview approach was used to guide the discussion. The transcribed discussions were analyzed. *Results* All four groups agreed that several facilitators are present, such as the unique position to reach women and the expertise in preventive health care. Identified barriers include unfamiliarity with interconception care among patients and health care providers, as well as lack of consensus about the concept of interconception care and how it should be organized. A broad educational campaign, local adaptation, and general agreement or a guideline for standard procedures were recognized as important for future implementation. *Conclusions* *for practice* This study identifies potentially important facilitators and barriers for the implementation of interconception care in Preventive Child Health Care services or comparable pediatric settings. These factors should be considered and strategies developed to achieve successful implementation of interconception care.

**Electronic supplementary material:**

The online version of this article (doi:10.1007/s10995-016-2046-5) contains supplementary material, which is available to authorized users.

## Significance

*What is already known on this subject?* Interconception care can contribute to the improvement of maternal and child health and the reduction of perinatal health disparities.

*What this study adds?* This study gives an overview of facilitators and barriers that should be considered when designing implementation strategies for interconception care, delivered to women who visit Preventive Child Health Care services with their children aged 0–4 years.

## Introduction

Interconception care, like preconception care, aims to improve pregnancy outcomes and thereby improve the health status of women and children. By offering care prior to pregnancy, the influence of potential risk factors for adverse pregnancy outcomes can be minimized. The advantages of providing this care before both first and subsequent pregnancies have already been demonstrated. There is growing evidence that preconceptionally delivered biomedical, psychosocial, and behavioral interventions are effective [1, 11, 18]. Furthermore, recent studies have shown a high prevalence of risk factors in the preconception and interconception period both in the Netherlands [21], as well as in the U.S. [12]. Despite consensus on the importance of preconception and interconception care, this care is still rarely delivered. Clear strategies to deliver this care are needed to guarantee potential health benefits [16, 17].

Recommendations for delivering interventions prior to pregnancy comprise a wide range of possibilities, including opportunistic utilization of health care visits [14]. This possibility is especially relevant to interconception care. Interconception care covers the period between pregnancies and is particularly valuable for women who have experienced an adverse pregnancy outcome [12]. Most women who give birth receive some form of perinatal care, postpartum care, and pediatric care for their newborn child. These visits therefore provide a meaningful gateway to interconception care, but they are generally not optimally utilized [3, 12].

In the Netherlands, interconception care is still uncommon as well. The opportunity to integrate interconception care in regular visits to either maternal or child care services deserves more attention. In maternal care provided by gynecologists, midwives, and family doctors, interconception care is currently applied on a small scale. However, the fact that there is no system of regular (e.g. annual) visits to these health care providers complicates the ability to reach women after the initial postpartum period. Alternatively, Preventive Child Health Care (PCHC) services offer the possibility to reach women who accompany their child to frequent well-child visits.

The Dutch PCHC services have some distinct features [5, 13, 22]. PCHC is organized nationally while delivered and financed on the municipal level. PCHC is provided by teams consisting of special trained PCHC physicians, nurses and physician assistants rather than pediatricians or family doctors. The latter two are only consulted in case of specific concerns. PCHC is offered for free to all families with children from birth through 19 years. It follows a standard set of visits based on the child’s age to monitor and promote optimal growth and development of the child. The care for 0–4 year-olds is organized in well-baby clinics with regular visits for vaccinations, screening and advice. These services have high (>95 %) attendance rates.

The frequent encounters with nearly all children and their parents in comparison with other health disciplines, make the PCHC services a valuable additional opportunity to embed interconception care in the Netherlands. This potential role for PCHC services in delivering interconception care has been recognized in a Dutch governmental advisory report on preconception care [8]. In addition, two recent studies acknowledged this opportunity and aimed at reaching women for advice on interconception care in well-baby clinics [2, 19]. Nevertheless, interconception care has not become standard care within PCHC services. Further exploration of the possibility of delivering interconception care through PCHC services is required.

Introducing interconception care in PCHC can be regarded as an innovative process which, within health care organizations, can be complex. In order for the innovation to be successful, it is essential to identify and consider important factors that facilitate or impede the proposed change [6]. Several reasons for poor delivery and uptake of interconception care have been described previously [7, 9, 12]. However, no analysis has been carried out of factors that could influence the introduction of interconception care in well child care in the Netherlands.

Using qualitative, focus group research methodology, we sought to learn more about the barriers and facilitators to integrating interconception care for mothers into PCHC services for children between 0 and 4 years of age.

## Methods

To learn more about integrating interconception care into well child visits, we used a qualitative approach based on focus group discussions with professionals [23]. We structured the study around a framework for determinants of innovation processes developed by Fleuren et al. [6]. This framework distinguishes four categories of determinants that can influence the four main stages of an innovation process: dissemination, adoption, implementation and continuation. These categories are (1) characteristics of the innovation, (2) characteristics of the professional, (3) characteristics of the organization, and (4) characteristics of the socio-political context. The categories are based on the identification of originally 50 potentially relevant determinants of innovation processes within health care organizations. We expected to find similar determinants in our study.

### Study Population

We identified four subgroups of professionals potentially involved in interconception care: (1) PCHC physicians, (2) PCHC nurses, (3) Health care professionals other than PCHC professionals who could provide interconception care (e.g. midwives, gynecologists, pediatricians, family doctors and occupational physicians), and (4) policymakers from local and national institutions concerned with interconception care or PCHC. For these subgroups we organized separate focus groups to minimize restraint in expressing opinions. We aimed to recruit 6–10 participants from different organizations, and with diverse experience with regards to interconception care for each group. We used different strategies to invite health care professionals to participate, including general invitations to organizations and personal invitations through contacts from a previous project [19].

### Data Collection

The four 3-h long meetings were held at a conference center in April 2015. Two researchers took turns guiding the discussions. Both researchers were present during all four meetings, as well as a third researcher conducting non-participant observation. The researchers took notes, and the sessions were also all recorded. The meeting started with a short introduction explaining the aim and assuring confidentiality. The discussion was set up as a semi-structured interview and was prompted with several statements that were sent to the participants in advance. These statements were chosen according to the determinants of the framework, i.e. statements regarding interconception care itself, interconception care for PCHC organizations and professionals, as well as the relationship with the socio-political context (see supplementary file). We chose not to give a firm definition of interconception care in advance, in order to stimulate the discussion on facilitators and barriers.

### Data Analysis

The focus group discussions were transcribed and sent to the participants to check for correctness unless a participant specifically requested not to be involved in this verification process. Names of participants were omitted from the transcripts. Instead, participant numbers were used to link participants with their statements. NVivo10 software (QSR International) was used to analyze the transcripts. A set of preliminary codes was developed from the notes and transcripts. This list was discussed between the researchers and adjusted during further analysis. The codes were structured to the concepts of determinants as described in the framework that was used. All themes were also coded to differentiate between facilitators and barriers. Coding was primarily performed by one researcher and verified by the other.

Ethical Statement: The qualitative study was reviewed by the Daily Board of the Medical Ethics Committee Erasmus MC as part of a larger study on implementation of interconception care in the Netherlands (MEC-2015-182). As a result of this review, the Board declared that the rules laid down in the Medical Research Involving Human Subjects Act (also known by its Dutch abbreviation WMO) do not apply to the study. No additional approval was requested for the current study since it is not based upon a clinical study or patient data.

## Results

### Study Population

A total of thirty-three participants took part in the focus groups. The characteristics of these participants are presented in Table 1. The participants came from different regions of the country and represented 24 different organizations. In order to gather diverse groups for the discussions, we started with a wide approach of inviting participants (N = 82). We approached several people from the same organization as we aimed to have at least one participant from that organization. Nineteen invitations received no response. Twenty-six people replied that they were unable to find space in their calendar, but they often tried to arrange a substitute instead. Four people were not interested.

**Table 1:** Characteristics of the participants

Characteristics	Group 1 n = 6	Group 2 n = 10	Group 3 n = 8	Group 4 n = 9
Profession	PCHC physician	PCHC nurse	Policymaker^a^	Health care professional other than PCHC^b^
Age (median, range)	41, 32–58	50, 38–59	53, 31–62	49, 31–61
Experience with preconception care/interconception care^c^				
Yes, as health care professional	1	2	0	8
Yes, as policymaker	0	0	5	1
Yes, as researcher	1	0	1	1
No experience	4	8	3	1
Organizations represented	5	6	8	9

^a^Policymakers were representatives of the professional organizations of midwives and PCHC physicians, the center of expertise for PCHC, a health insurance company, Municipal Health Services (2), the Commission for Perinatal Health, and management bodies of PCHC organizations. This included participants with a background as a midwife, PCHC physician, PCHC nurse and preconception care researcher

^b^Family doctors (3), Midwives (2), Gynecologist (1), Pediatricians (2) and Occupational Physicians (1)

^c^Numbers can add up to more than the total number of participants due to experience in different fields

### Facilitators and Barriers for the Implementation of Interconception Care

We identified a wide range of facilitators and barriers as described in Table 2. Topics that were mentioned in at least two groups were included.

**Table 2:** Facilitators and barriers for implementation of interconception care in PCHC services

Categories of determinants	Facilitators	Barriers
*Characteristics of interconception care*		
Appreciation of concept	Repetition of message via opportunities with target audience and various media Systematic general approach	Unfamiliarity with concept Lack of consensus on meaning and use of the concept
Applicability	Tools, guidelines for care Option to offer care (1,2) Clear evident general advice Receptive period (1,4) Personal approach	Different backgrounds and needs Complex individual care Sensitive topic (1,2,4)
*Characteristics of the (PCHC) professional*		
Competence and self efficacy	Training/education Link task to current expertise Familiarity with families (2,4)	New knowledge required Uncertainty about self-efficacy (2,4)
Attitude and expectations	Benefits for child in care, parents and future child (1,2,4)	Concern about response and cooperation (2,4) Concern about feasibility
*Characteristics of the (PCHC) organization*		
Organizational structure	Overall support in organization (2,3,4)	Complex decision making process (2,3,4) Organizational differences
Organizational expertise	Accessible care with high coverage of target population Preventive care (including pre-natal)	Focus on child care (separated from maternal care) (3,4)
Reimbursement	Providing insight in advantages	Costs of time and staff investment
Logistical procedures	Local solutions for unavailable standard procedures (2,3,4)	Lack of suitable administration, planning and referral system (2,3,4)
*Characteristics of the context*		
Regulations and legislation	National guideline for PCHC Exploring health insurance options Assuring continuation	Dependency on local priorities Overlap of different health care and reimbursement systems
Societal relevance	Awareness of perinatal health Attention for preventive measures	Changes in organization of child care
Collaboration between professions	Good cooperation and agreements on responsibilities	Lack of arrangements or structural contact (1,3,4)

The presented facilitators or barriers were identified in all four focus groups unless otherwise stated by numbering the relevant focus groups behind the specific facilitator or barrier

### Characteristics of Interconception Care

In all the discussions, unfamiliarity with the concept of interconception care was brought forward as an important impeding issue for both parents and health care professionals. Participants thought that a widespread approach was required to inform people of interconception care repeatedly and not just on one occasion. They mentioned using the following opportunities: community gatherings, the internet, popular television shows, and integration in existing health care and education programs. It was argued that interconception care has to become ‘normal’ to both health care providers and the general population. Accordingly, interconception care should be provided systematically to everyone instead of exclusively to high risk groups. Another barrier was a lack of consensus on aspects of interconception care such as the terminology, the definition, the content, the implementation and the target audience.

Evidence-based guidelines for the provision of interconception care would enhance the ability of PCHC providers to offer services to new mothers. Participants suggested that mothers who are considering having another child are receptive to information that would improve the well-being of their future baby. To obtain high compliance, the use of incentives and a reminder system for appointments was suggested. A personal approach was thought to be important. The complexity of applying interconception care was stressed as well. Providers must deal with factors such as different individual backgrounds, medical needs and social needs, as well as challenging aspects of the content (e.g. behavioral change and discussing a desire to become pregnant). However, others pointed out that this complements PCHC professionals’ expertise.

### Characteristics of the Preventive Child Health Care Professional

Current expertise of PCHC professionals is in part closely linked to aspects of interconception care. These skills include giving preventive advice, motivational interviewing and dealing with sensitive topics (e.g. social needs). There are also limitations to the competence of PCHC professionals with respect to interconception care since their professional focus is preventive health care for children and not for women. Even with extra training, doubt was expressed by both PCHC staff as well as other professionals about dealing with the mother’s medical care, such as chronic disease and obstetric complications. On the other hand, PCHC professionals are often familiar with individual family backgrounds and needs because of regular child visits. This relationship is an advantage, but concerns about harming this relationship might impede the fulfillment of interconception care. Concerns included fear of stigmatizing and creating a sense of guilt and not being able to respect personal choices. At the same time, all the professionals acknowledged the health benefits of applying interconception care.

### Characteristics of the Preventive Child Health Care Organization

The participants recognized that an innovation like interconception care within PCHC organizations can be challenging because of a multiple tier system, which consists of an internal management structure closely tied to local and national government structures. In addition, PCHC health care professionals also clearly wanted to be involved in the introduction of any innovation. A uniform national implementation strategy would be complex since PCHC organizations differ in terms of size, personnel management, organization of care and specific focus areas. Regardless, the following common factors between these organizations were mentioned as facilitators for interconception care: (1) the regular and accessible form of care which covers and reaches almost the whole population with young children; and (2) the preventive aspect of this care for optimal child development, which often includes a form of prenatal education.

Given that maternal care is not part of PCHC expertise there are logistical and financial challenges, according to the participants. The participants also mentioned facilitating factors: (1) a current shift in care from the child only, towards the child including his/her context, the family; (2) the interpretation of interconception care as care for a future child, which fits in with the preventive health care task for children; (3) integration of interconception care in current appointments; and (4) local solutions to logistical challenges if possible. In some places such solutions already exist regarding the availability of a client medical record for an unborn child. With respect to the financial challenges, it was stressed that sufficient resources for interconception care are essential. Promoting the advantages and necessity of interconception care could help to acquire these resources.

### Characteristics of the Context

Arranging a sustainable financial compensatory system has several challenges but was considered to be important. A uniform national policy would be helpful to allow for reimbursement by local municipalities. Currently, PCHC organizations have to negotiate for reimbursement of extra care that is not covered by the national policy and are then dependent on local priorities regarding health care expenses. Several participants saw coverage by health insurance companies as an option, but this form of reimbursement is still uncommon for PCHC. Reorganization of child care within municipalities was seen as a potential opportunity for innovations in PCHC, but mainly judged as a potential limitation because of the uncertainty it implicates. Other facilitating factors mentioned include current societal attention for improvement of perinatal health and general preventive measures such as a healthy diet and lifestyle. Lastly, improvement of cooperation between health care providers was brought forward as a determinant for interconception care. Aspects such as regular contact, and clear agreements between different health care providers on responsibilities for interconception care were seen as valuable.

### Interpretation of the Concept of Interconception Care

Several common interpretations of the content and implementation of interconception care were identified. Regarding the content, most aspects of preconception care were mentioned for interconception care with additional attention to contraceptive counselling. With respect to the target audience, most participants argued for a broad general approach including mothers and their partners. Opinions on the timing of interconception care differed. Some participants thought interconception care could start at the first postpartum visit, but others thought people may not be receptive at this stage and suggested 6 months. A year postpartum was argued to be too late. Repeatedly giving information and following up on this in a flexible manner, accounting for individual parental needs, was considered a good approach.

All the health care providers acknowledged their responsibility for interconception care to some extent. Some of the policymakers debated the responsibilities of PCHC services regarding this form of preventive care.

## Discussion

### Main Findings

During the focus groups, many aspects were discussed regarding implementing interconception care for women in PCHC services for 0–4 year-olds in the Netherlands. All four groups appreciated the benefits of implementing interconception care in Dutch PCHC services, utilizing their unique position, which brings them into contact with almost all young children and their mothers, as well as their expertise in preventive health care. Participants also suggested informing the general public about interconception care, training professionals, and creating local as well as (inter)national agreement on how to organize and reimburse interconception care. The responsibility of many related professionals and public health or governmental bodies was considered of great importance in facilitating the implementation of interconception care.

### Comparison to the Literature

Our results reflect opportunities and barriers mentioned in the literature on preconception and interconception care. Concerns about the complexity of delivering interconception care are seen in studies in the U.S. such as described by Handler et al. [7]. Their study of two community high risk interconception care programs demonstrated that interconception care is ‘a complex process of matching interventions and services to meet women’s unique needs, including their socioeconomic needs’. They also described the importance of educating both women and health care providers about the benefits of this care. Hogan et al. [9] found that even when common barriers were actively removed, such as provision of transportation, childcare and free service, no consistent participation could be obtained for their interconception intervention aimed at vulnerable women. On the other hand, although it did not meet their aims, they did reach an average overall participation rate of 52 % with their approach. Their analysis did not yield clear influencing factors. Velott et al. [20] described the advantages of combining active and passive recruitment techniques, including partnering with local community organizations for the recruitment of hard-to-reach women.

These studies all targeted high risk communities. In our discussions, a general standard care approach including low risk groups was preferred. To utilize every office visit as a potential educational opportunity for interconception counseling and discussing a personal reproductive life plan has been advocated before with “every woman every time” [3]. Although ideally a full package of health and social interventions would be delivered to all women and couples of reproductive age everywhere, interventions often need to be tailored to local realities as argued by Mason et al. [15] for low and middle income countries (LMIC). The challenge of organizing this preventive care for it to be fully realized is not confined to LMIC. To let preconception and interconception care become part of routine care, the need for policies, a reimbursement system and the empowerment of staff is clear [1, 10, 17].

We structured the analysis according to an existing framework which originally listed 50 potentially relevant determinants of innovation processes in four identified categories [6]. Later work, based on a combination of expert consultations and empirical studies in schools, PCHC and health promotion programs, modified the list to 29 determinants [4]. We identified many determinants consistent with this list (e.g. content awareness, procedural clarity, expectations, relevance, social support, and aspects related to competence, regulation, the client, and the organization). However, determinants such as replacement of staff, a coordinator, and information on use of the innovation did not appear in our analysis. An explanation could be that these determinants are more essential in a stage when the innovation is already in use; the stage of continuation. Similarly in our study, assuring continuation of interconception care instead of limited project based implementation was recognized as an important facilitating determinant.

### Strengths and Limitations

The interaction within the focus groups helped to gain a comprehensive overview of determinants from different perspectives. When interpreting these results, certain limitations should be taken into account such as the relatively small sample size and the influence of potential bias. Although the sample size per group was small, we did obtain our stated aims for each group: a minimum of six participants, a mixture of different levels of experience with interconception care, and representation of the targeted disciplines, various organizations and regions. Therefore, we believe that the sample of professionals was a good reflection of the range of potential stakeholders. Bias may have resulted from a sample of participants who were interested in interconception care, as well as moderators who had prior interest in the research topic.

In addition to these limitations, this study was primarily based on professionals’ expectations, rather than actual experiences. If interconception care were to be implemented in PCHC, this could be a focus of future research. Future research could also include client perspectives.

### Practical Implications

This study applies specifically to PCHC services in the Netherlands, but the results could also be valuable to other health care settings that may play a role in interconception care. The opportunity to implement some form of interconception care for women in PCHC services was recognized by most participants. However, they also had clear reservations about what form and to what extent interconception in PCHC services could be offered. This justifies further evaluation of different possibilities for actual implementation in the Netherlands. We recommend targeting the identified facilitators and barriers within implementation strategies to achieve successful integration of interconception care in PCHC services, and seizing this opportunity to integrate health promotion for women and children in routine postpartum care.

## Electronic supplementary material

Below is the link to the electronic supplementary material.

Supplementary material 1 (XLSX 10 kb)

## References

[1] Atrash H, Jack BW, Johnson K (2008). Preconception care: A 2008 update. Current Opinion in Obstetrics and Gynecology.

[2] de Smit DJ, Weinreich SS, Cornel MC (2015). Effects of a simple educational intervention in well-baby clinics on women’s knowledge about and intake of folic acid supplements in the periconceptional period: A controlled trial. Public Health Nutrition.

[3] DeCesare JZ, Jackson JR, Phillips B (2015). Interconception care opportunities for mom and baby. Obstetrical & Gynecological Survey.

[4] Fleuren M, Paulussen TG, Van Dommelen P, Van Buuren S (2014). Towards a measurement instrument for determinants of innovations. International Journal for Quality in Health Care.

[5] Fleuren M, van Dommelen P, Dunnink T (2015). A systematic approach to implementing and evaluating clinical guidelines: The results of fifteen years of Preventive Child Health Care guidelines in the Netherlands. Social Science and Medicine.

[6] Fleuren M, Wiefferink K, Paulussen T (2004). Determinants of innovation within health care organizations: Literature review and Delphi study. International Journal for Quality in Health Care.

[7] Handler A, Rankin KM, Peacock N, Townsell S, McGlynn A, Issel LM (2013). The implementation of interconception care in two community health settings: Lessons learned. American Journal of Health Promotion.

[8] Health Council of the Netherlands. (2007). *Preconception care: A good beginning. The Hague: Health Council of the Netherlands. Publication no. 2007/19E*.

[9] Hogan, V. K., Amamoo, M. A., Anderson, A. D., Webb, D., Mathews, L., Rowley, D., & Culhane, J. F. (2012). Barriers to women’s participation in inter-conceptional care: A cross-sectional analysis. *BMC Public Health, 12*((Hogan V.K.) Gillings School of Global Public Health, University of North Carolina at Chapel Hill, Chapel Hill, NC, USA.), 93. 10.1186/1471-2458-12-93 PMC3317822 22296758

[10] Jack BW, Atrash H, Bickmore T, Johnson K (2008). The future of preconception care: A clinical perspective. Womens Health Issues.

[11] Jack BW, Atrash H, Coonrod DV, Moos MK, O’Donnell J, Johnson K (2008). The clinical content of preconception care: An overview and preparation of this supplement. American Journal of Obstetrics and Gynecology.

[12] Johnson KA, Gee RE (2015). Interpregnancy care. Seminars in Perinatology.

[13] Kuo AA, Inkelas M, Lotstein DS, Samson KM, Schor EL, Halfon N (2006). Rethinking well-child care in the United States: An international comparison. Pediatrics.

[14] Lassi ZS, Dean SV, Mallick D, Bhutta ZA (2014). Preconception care: Delivery strategies and packages for care. Reproductive Health.

[15] Mason E, Chandra-Mouli V, Baltag V, Christiansen C, Lassi ZS, Bhutta ZA (2014). Preconception care: Advancing from ‘important to do and can be done’ to ‘is being done and is making a difference’. Reproductive Health.

[16] Shannon GD, Alberg C, Nacul L, Pashayan N (2014). Preconception healthcare delivery at a population level: Construction of Public Health Models of Preconception Care. Maternal and Child Health Journal.

[17] Shawe J, Delbaere I, Ekstrand M, Hegaard HK, Larsson M, Mastroiacovo P, Tyden T (2015). Preconception care policy, guidelines, recommendations and services across six European countries: Belgium (Flanders), Denmark, Italy, the Netherlands, Sweden and the United Kingdom. The European Journal of Contraception & Reproductive Health Care.

[18] Temel S, van Voorst SF, de Jong-Potjer LC, Waelput AJ, Cornel MC, de Weerd SR, Steegers EA (2015). The Dutch national summit on preconception care: A summary of definitions, evidence and recommendations. Journal of Community Genetics.

[19] van Voorst SF, Vos AA, de Jong-Potjer LC, Waelput AJ, Steegers EA, Denktas S (2015). Effectiveness of general preconception care accompanied by a recruitment approach: Protocol of a community-based cohort study (the Healthy Pregnancy 4 All study). BMJ Open.

[20] Velott DL, Baker SA, Hillemeier MM, Weisman CS (2008). Participant recruitment to a randomized trial of a community-based behavioral intervention for pre- and interconceptional women findings from the Central Pennsylvania Women’s Health Study. Womens Health Issues.

[21] Vink-van Os LC, Birnie E, van Vliet-Lachotzki EH, Bonsel GJ, Steegers EA (2015). Determining pre-conception risk profiles using a national online self-reported risk assessment: A cross-sectional study. Public Health Genomics.

[22] Wieske RC, Nijnuis MG, Carmiggelt BC, Wagenaar-Fischer MM, Boere-Boonekamp MM (2012). Preventive youth health care in 11 European countries: An exploratory analysis. International Journal of Public Health.

[23] Wong LP (2008). Focus group discussion: A tool for health and medical research. Singapore Medical Journal.

